# Exploring the PDZ, DUF, and LIM Domains of Pdlim5 in Dendrite Branching

**DOI:** 10.3390/ijms25158326

**Published:** 2024-07-30

**Authors:** Yogesh Srivastava, Maxsam Donta, Lydia L. Mireles, Adriana Paulucci-Holthauzen, Leilei Shi, Mark T. Bedford, M. Neal Waxham, Pierre D. McCrea

**Affiliations:** 1 Department of Genetics, University of Texas MD Anderson Cancer Center, Houston, TX 77030, USA; 2 Program in Genetics & Epigenetics, University of Texas MD Anderson Cancer Center, UT Health GSBS, Houston, TX 77030, USA; 3 Department of Neurobiology & Anatomy, University of Texas MD Anderson Cancer Center, UT Health GSBS, Houston, TX 77030, USA; 4 Department of Epigenetics & Molecular Carcinogenesis, University of Texas MD Anderson Cancer Center, Houston, TX 77030, USA; 5 Program in Neuroscience, University of Texas MD Anderson Cancer Center, UT Health GSBS, Houston, TX 77030, USA

**Keywords:** neuron, dendrite, shape, morphology, cytoskeleton

## Abstract

The branched architecture of neuronal dendrites is a key factor in how neurons form ordered networks and discoveries continue to be made identifying proteins and protein–protein interactions that direct or execute the branching and extension of dendrites. Our prior work showed that the molecular scaffold Pdlim5 and delta-catenin, in conjunction, are two proteins that help regulate the branching and elongation of dendrites in cultured hippocampal neurons and do so through a phosphorylation-dependent mechanism triggered by upstream glutamate signaling. In this report we have focused on Pdlim5’s multiple scaffolding domains and how each contributes to dendrite branching. The three identified regions within Pdlim5 are the PDZ, DUF, and a trio of LIM domains; however, unresolved is the intra-molecular conformation of Pdlim5 as well as which domains are essential to regulate dendritic branching. We address Pdlim5’s structure and function by examining the role of each of the domains individually and using deletion mutants in the context of the full-length protein. Results using primary hippocampal neurons reveal that the Pdlim5 DUF domain plays a dominant role in increasing dendritic branching. Neither the PDZ domain nor the LIM domains alone support increased branching. The central role of the DUF domain was confirmed using deletion mutants in the context of full-length Pdlim5. Guided by molecular modeling, additional domain mapping studies showed that the C-terminal LIM domain forms a stable interaction with the N-terminal PDZ domain, and we identified key amino acid residues at the interface of each domain that are needed for this interaction. We posit that the central DUF domain of Pdlim5 may be subject to modulation in the context of the full-length protein by the intra-molecular interaction between the N-terminal PDZ and C-terminal LIM domains. Overall, our studies reveal a novel mechanism for the regulation of Pdlim5’s function in the regulation of neuronal branching and highlight the critical role of the DUF domain in mediating these effects.

## 1. Introduction

Neurons are the most architecturally complex cells in the body, and much of this complexity stems from the elaborate dendritic arbors that emanate from neuronal soma [1]. Dendrites are the major sites for receiving inputs in connected neuronal networks, and their proper formation and stability are essential for normal brain function [1,2]. The formation of dendritic arbors requires mechanisms to regulate both their elongation and branching and involves an intricate coordination of local modulation of the actin cytoskeleton [3,4]. A variety of actin regulatory proteins are necessary for the coordination of branching and elongation [4]. Among these regulators, the PDLIM family of proteins is one class of molecules essential for integrating aspects of actin growth and branching and accomplishes these functions through a multi-domain architecture [5,6]. Reading from its N- to C-terminus, Pdlim5 is made up of three principal domains, PDZ (postsynaptic density 95, discs large, and zonula occludens-1), DUF4749 (Domain of Unknown Function), and a trio of LIM domains (LIN-11, Isl-1, and MEC-3) [6,7]. The PDZ and LIM domains are ubiquitous amongst the seven members of the Pdlim family of proteins, although Pdlim5, 6, and 7 have a triplet of LIM domains at their C-terminus while Pdlim1-4 have a single LIM domain [5]. Unique molecular partners have been identified to associate with each of these domains. For example, the PDZ domain supports direct interactions with the actin-binding protein α-actinin [8], while the LIM domains localize the protein to the actin cytoskeleton by yet unknown mechanisms [5]. These interactions have been well characterized in actin-rich stress fibers and the Z-disc of muscle fibers [7,9]; however, their interactions with the neuronal actin cytoskeleton are less well characterized. Interestingly, Pdlims have been identified to influence the trafficking of growth factor receptors [10,11,12], the nicotinic acetylcholine receptor [13], and the GluR1 subunit of ionotropic glutamate receptors [14] in neuronal membranes through involvement in the endosomal recycling pathway that relies on the actin cytoskeleton [15]. In the context of developing neurons, we have previously shown that Pdlim5 is a critical component of a delta-catenin-mediated switching mechanism that controls dendritic branching [16,17]. Phosphorylation of delta-catenin’s PDZ domain switches binding from Magi1 (a member of the MAGUK family of PDZ-containing proteins) to Pdlim5, and this switch appears necessary for the initiation of dendrite branch formation, presumably via locally regulating the actin cytoskeleton [16]. We further showed that Pdlim5’s LIM domain interacts with PalmD, a protein important in initiating dendritic branching, and that Pdlim5 and PalmD influence branching in a way that is functionally interdependent [18]. These results point to Pdlim5 as a critical protein for coordinating the molecular machinery necessary to establish a properly formed dendritic arbor, and it is thus unsurprising that Pdlim5 dysregulation has been proposed as a factor contributing to a variety of human neurodevelopmental diseases.

These earlier studies identified a critical role for Pdlim5 in regulating dendritic branching and further identified molecular partners that, in part, mediate these effects. However, there remains much to learn about the domain structure of Pdlim5 and how individual domains participate in producing a coordinated set of binding interactions necessary to initiate dendritic branching. In this report, we directly investigate the role of the PDZ, DUF, and LIM domains of Pdlim5 and for the first time identify the dominant role of the DUF domain as an essential region critical for affecting dendritic branching. We further show that there is a direct interaction between the PDZ domain and the C-terminal LIM domain of Pdlim5 and propose that intra-molecular binding between these two domains is part of the regulatory mechanism for controlling Pdlim5 function. This allosteric autoregulatory mechanism appears to be a common feature of a variety of actin-binding proteins [19]. Interestingly, autoinhibition is relieved by one or more ligand-binding events and/or phosphorylation of one or multiple domains that ultimately influence different active states of the actin-binding proteins. In this regard, we identified that a specific Tyr residue within the DUF domain of Pdlim5 (Y251) plays an additional regulatory role in dendritic branching. These results identify Pdlim5 as a key integrator for protein–ligand binding and protein tyrosine kinase signaling that mediates essential steps in controlling dendrite branching.

## 2. Results

### 2.1. Impact of Expressing Isolated Pdlim5 Domains upon Dendrite Morphology

**Figure 1:**
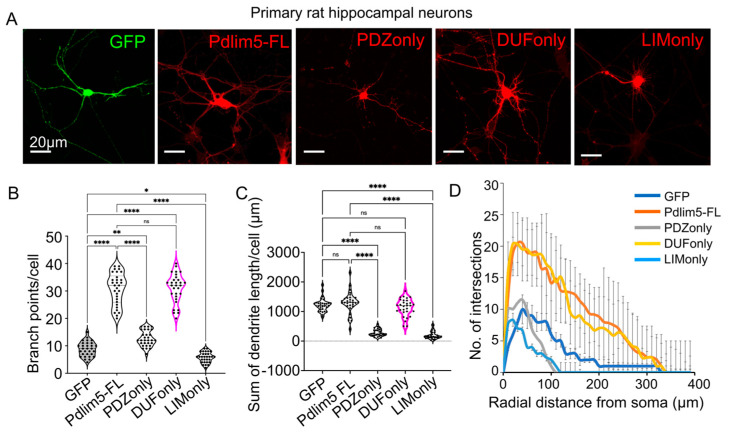
Impact of expressing isolated Pdlim5 domains upon dendrite morphology. (**A**) Representative immunofluorescent images of primary hippocampal neurons following transfection with the indicated constructs (GFP, Pdlim5 full-length (FL), PDZ only, DUF only, or LIM only, isolated domains). Neurons were transfected at DIV3 post-plating and were fixed at DIV7. Each construct carried a Myc tag that was detected using immunofluorescence labeling and image quantification as detailed in the methodology. Scale bars in the images represent 20 µm. (**B**) Quantitation of the average number of branch points per cell following exogenous expression of Pdlim5 isolated domains. The data were collected from ≥10 neurons from each of three biological replicates, with each dot representing data from one neuron. * Indicates significance ≤ 0.02, ** indicates significance ≤ 0.002, and **** indicates significance ≤ 0.0001 while ns shows non-significant from ANOVA analysis. (**C**) Quantitation of the average dendrite length per cell (in mm) following expression of Pdlim5 isolated domains was determined for the same dataset used for branch point analysis in panel (**B**). The statistical significance, determined using one-way ANOVA, is indicated as **** at *p* ≤ 0.0001. (**D**) Sholl analysis of neurons expressing the following constructs: GFP (dark blue), full-length Pdlim5 (Pdlim5 FL, orange), and isolated domains of Pdlim5 (PDZ only, grey; DUF only, yellow; or LIM only, cyan). Data points are the average values (*n* ≥ 15 neurons from three biological replicates), with error bars indicating SEM. The significance was assessed using a two-way ANOVA (mixed-effect model for missing values and including repeated measures) with Bonferroni post hoc analysis that varies from *p* ≤ 0.0001 to *p* < 0.05, based on radial distance from the cell soma (compared to cells expressing GFP alone and Pdlim5-FL).

One of our study’s goals was to clarify the contributions of Pdlim5’s principal domains to dendritic morphology. The basic domain organization of Pdlim5 includes, from N- to C-terminus, the PDZ domain, the DUF domain, and a triplet of LIM domains. For this purpose, constructs of Pdlim5’s isolated domains were expressed starting at 3 days in vitro (DIV) in rat hippocampal neurons, and dendritic growth was assessed on day 7 (Figure 1A). GFP expression alone served as a negative control, while full-length Pdlim5 served as a positive control. To facilitate detection, each construct was myc-tagged, and transfected cells were identified by immunolabeling. The numbers of dendritic tips as well as dendritic lengths were subjected to quantitation. In contrast to the PDZ and LIM domains, our findings pointed prominently toward the isolated DUF domain as the region responsible for increasing dendritic branching (Figure 1B). In fact, the DUF domain itself produced an equivalent increased branching to that of full-length Pdlim5 (Figure 1B). Dendrite lengths and branching were reduced upon PDZ or LIM expression (Figure 1B,C), suggesting there might be a subtle dominant negative effect of these domains, while there was little impact of the isolated DUF domain upon lengths relative to negative-control GFP or full-length Pdlim5 (Figure 1C). Positive effects of Pdlim5 expression upon branching, as opposed to lengthening, are consistent with our earlier findings [16,18]. Furthermore, the dendritic complexity was evaluated using Sholl analysis. In keeping with the above-noted counting of branch points per cell, Sholl analysis indicated that neurons expressing the isolated DUF domain (or the positive control full-length Pdlim5) exhibited higher dendritic complexity than negative-control GFP or the isolated PDZ or LIM domains (Figure 1D). In fact, in contrast to DUF, Sholl analysis indicated that dendritic complexity was reduced in neurons that expressed isolated PDZ or LIM domains (each relative to GFP negative control and positive control Pdlim5-FL), again consistent with a potential dominant negative effect (Figure 1D). A dominant negative effect remains speculative, however. Efforts to first knock down endogenous Pdlim5 followed by rescue with the isolated domains were overly toxic in the neuronal cultures, preventing this assessment. As further examined and supported below, these findings are consistent with the possibility that Pdlim5’s DUF domain contains the biologically relevant activity for increasing dendritic branching to support the formation of neuronal networks.

### 2.2. Domain Deletions of Pdlim5 Support the Importance of the DUF Domain in Dendrite Branching

**Figure 2:**
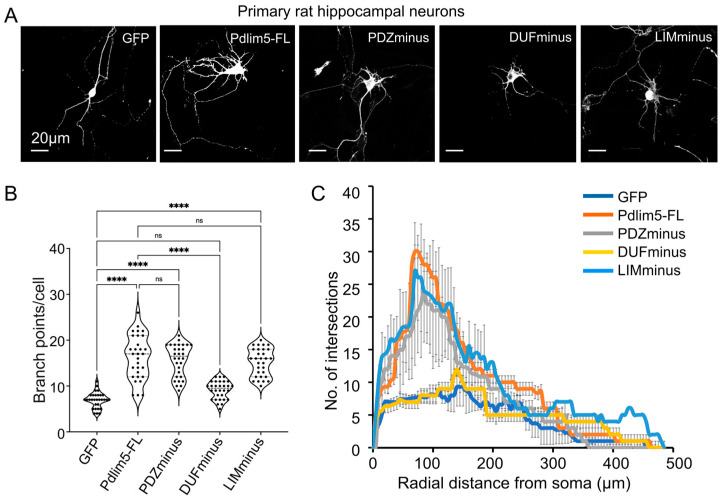
Domain deletions of Pdlim5 support the importance of the DUF domain in dendrite branching. (**A**) Representative images of neuronal phenotypes following expression of Pdlim5 domain-deletion constructs. Neurons were transfected at DIV3 and fixed at DIV7. The images are shown in binary intensity to highlight differences in the impact of the deletion constructs on morphology. Fluorescent images were produced either from GFP (as control) or following immunolabeling with antibodies to the Myc-tagged full-length or deletion constructs of Pdlm5. Scale bars are 20 mm. (**B**) Quantitation of the average number of branch points per cell following expression of domain deletion constructs (PDZminus, DUFminus, or LIMminus). The data were collected from ≥10 neurons, with each dot representing one neuron from each of three biological replicates. The violin plot quartiles (dotted lines) indicate data ranges where the middle dark line represents the median, and statistical significance determined using one-way ANOVA is indicated as *p* ≤ 0.0001 (****), ns (not significant). (**C**) Sholl analysis of neurons expressing the following constructs: GFP (dark blue), full-length Pdlim5 (Pdlim5 FL, orange), and deletion constructs of Pdlim5 (PDZminus, grey; DUFminus, yellow; or LIMminus, cyan). Data points are the average values (*n* ≥ 15 neurons from three biological replicates) with error bars indicating SEM. The significance was assessed using a two-way ANOVA (mixed-effect model for missing values and including repeated measures) with Bonferroni post hoc analysis that varies from *p* ≤ 0.0001 to *p* < 0.05, based on radial distance from the cell soma (compared to cells expressing GFP alone and Pdlim5-FL).

To complement our findings from the expression of isolated Pdlim5 domains, a series of Pdlim5 deletion constructs that systematically remove one domain at a time were similarly expressed in DIV3 neurons and analyzed at DIV7 (Figure 2A). We refer to these myc-tagged constructs as PDZminus, DUFminus, and LIMminus. Full-length Pdlim5 serves as our positive control, with our negative control being the expression of GFP alone. Notably, only the removal of the DUF domain produced a significant decrease in dendritic branching (Figure 2A,B), nearly returning to the effects of GFP expression alone (negative control). The PDZminus or LIMminus constructs produced branching not statistically different from full-length Pdlim5 (Figure 2B). These findings were also evident in Sholl analyses performed to evaluate morphologic alterations following the expression of domain-deletion constructs (Figure 2C). Together, our isolated-domain and domain-deletion findings consistently suggest a role of Pdlim5’s DUF domain in promoting dendritic branching.

### 2.3. Comparison of the DUF Domain between Species and a Potential Role for Phosphorylation at Residue Y251

**Figure 3:**
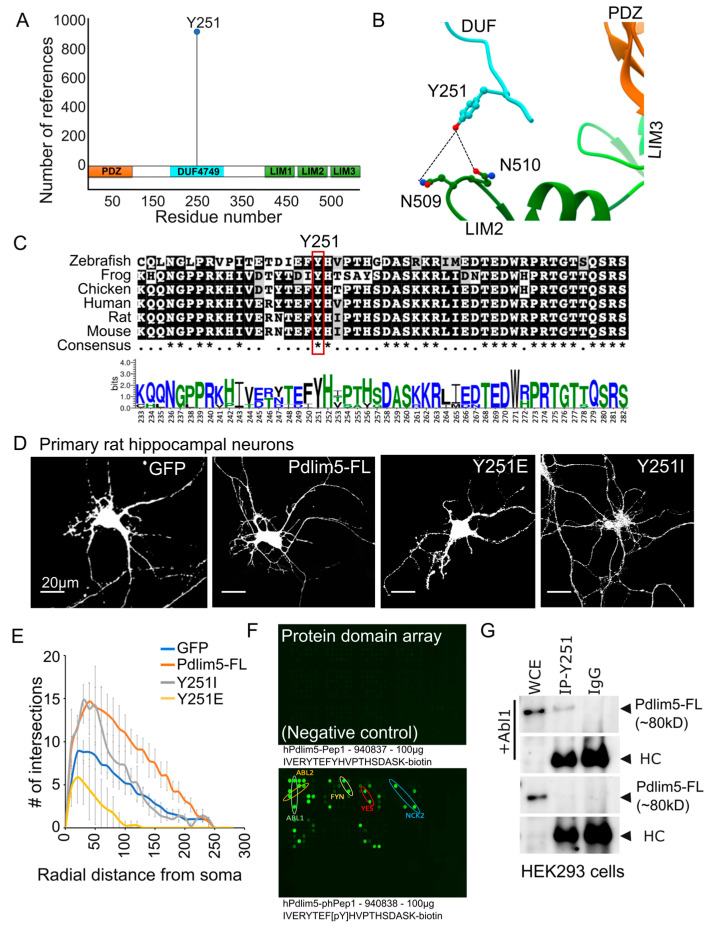
DUF domain cross-species conservation and phosphorylation at residue Y251. (**A**) Figure from PhosphoSitePlus depicting Pdlim5’s three principal domains, highlighting the phosphorylation of Y251 in the central DUF domain. The X axis indicates amino acid residue positions, and the Y axis represents the total number of references available on the PhosphoSitePlus website. (**B**) AlphaFold modeling of Pdlim5 with a focus on Y251 in the central DUF domain. Within the larger Pdlim5 folded structure, AlphaFold predicts the proximity of Y251 to two asparagine residues in the LIM2 domains. (**C**) Amino acid sequence alignment indicates the conservation of Y251 across vertebrate species, as well as the conservation of the closely surrounding part of the larger DUF domain. (**D**) Binary images of primary rat hippocampal cells following the expression of the point mutants Y251E or Y251I, relative to the expression of GFP or Pdlim5. Results show a significant decrease in dendritic branching upon expression of Y251E, while Y251I exhibited a more modest effect. (**E**) X-axis represents radial distance from soma in micrometer (µm) while Y axis shows number (#) of intersection. Sholl analysis quantifying the impact of the Y251E or Y251I mutants on neurite morphological characteristics. At least 15 neurons from three biological replicates were consolidated for the Sholl analysis. The significance was assessed using a two-way ANOVA (mixed-effect model for missing values and including repeated measures) with Bonferroni post hoc analysis that varies from *p* ≤ 0.0001 to *p* < 0.05, based on radial distance from the cell soma (compared to cells expressing GFP alone and Pdlim5-FL). Scale bars in the images represent 20 µm. (**F**) Data from a SH2 peptide domain array from proteins that are known to have selective capacities to bind phospho-residues. The array, described in the methods, was probed with DUF domain peptides surrounding the Y251 phosphorylation site. The top panel shows the binding profile of the unphosphorylated peptide-IVERYTEFYHVPTHSDASK-biotin, and the bottom panel shows the binding profile of the phosphorylated peptide-IVERYTEF[pY]HVPTHSDASK-biotin. Bound peptides were detected with streptavidin-Cy3. (**G**) Immunoblot of immunoprecipitates from HEK-293 cells expressing exogenous Abl1 kinase (top panel) versus not expressing exogenous Abl1 (lower panel). Antibodies selectively directed against phospho-Y251 within Pdlim5 were used for the initial pull-down followed by immunoblotting for Pdlim5.

Given the results identifying the important role of Pdlim5’s DUF domain in dendrite branching, we surveyed existing databases to assess its conservation across species and to assess whether phospho-residues with potential regulatory relevance stood out (Figure 3). Indeed, Y251 in the core DUF domain of Pdlim5 appears significantly in proteomic investigations curated in PhosphoSitePlus (Figure 3A). For additional context, we modeled the region including Y251 using AlphaFold (Figure 3B) which predicts a potential interaction with Asn 509 and 510 in the second LIM domain.

Further, Y251 along with the larger DUF domain is highly conserved across vertebrates (Figure 3C), supporting its potential importance in regulating Pdlim5’s functions. To assess the potential functional relevance of Y251 in the regulation of dendritic branching, we created two mutants of Y251, namely Y251I and Y251E. While Y251I (isoleucine substitution) would be anticipated to prevent phosphorylation, the glutamate substitution (here in Y251E) would instead potentially mimic phosphorylation by introducing a negative charge. We acknowledge that a glutamate replacement is often considered a poor mimic of tyrosine phosphorylation (likewise the case for an aspartate substitution) [20]. Nonetheless, one can regard both mutants as attempts to probe the general relevance of Y251 in DUF-domain functions in dendrite branching. Panel 3D shows binary images following the expression of Y251E and Y251I mutants in rat hippocampal neurons, relative to the expression of GFP (negative control) or Pdlim5 (positive control) as done for evaluating domain impacts on dendrite branching (Figure 1 and Figure 2). Relative to wild-type Pdlim5, Y251E expression resulted in considerably decreased dendritic length and branching (Figure 3D,E). Interestingly, Y251I showed little effect on branches close to the cell body but had a significant negative effect on those more distal (Figure 3E). This suggests that the regulation of branching is not affected, but the extension of dendrites is. It is not immediately obvious why the Y251I mutant would produce such effects, and future studies will be required to address this finding more fully. Regardless, these findings appear consistent with the hypothesis that Y251 is a functionally relevant residue and that its phosphorylation may regulate Pdlim5 effects. More speculatively, if Y251E partially mimics phospho-Y251, then our findings suggest that a phospho-switch exists at Y251 that negatively impacts Pdlim5’s branching function in dendrites (Figure 3E).

Finally, to further assess the potential functional relevance of Y251, we employed an array representing protein domains having the potential to selectively bind phospho-tyrosine in accordance with the surrounding sequence context (e.g., SH2, PTB, PH, HYB, and C2 domains). Specifically, we probed the array with two Pdlim5 DUF-domain peptides of the same amino acid sequence (Uniprot ID Q96HC4; human residues 242–261) but differing in their phospho-status at the central Y251 residue (phospho-peptide obtained via commercial synthetic chemistry with a phosphate group attached at Y251). Strikingly, while the unphosphorylated DUF peptide showed little to no interactions with the array, the phospho-Y251 peptide bound selectively to several protein kinase SH2 domains (Figure 3F). While requiring direct validation, a number of these kinases might be relevant to phosphorylating Y251 within Pdlim5’s DUF domain and thereby contribute to a Pdlim5-dependent function in modulating dendritic shape and complexity. Based on the peptide array data identifying Abl1 as a potential kinase phosphorylating Y251, we expressed Pdlim5 with and without Abl1 in HEK293 cells and immunoprecipitated Pdlim5 with a Y251 phospho-specific antibody (Figure 3G; see also full-length blot in Figure S2). Consistent with the above-referenced proteomic studies, our data likewise suggests that Y251 is a bona fide phosphorylation site, and potentially is a direct substrate for Abl1. These findings open the door for future studies to confirm the kinases responsible for Y251 phosphorylation and to assess their roles in regulating Pdlim5 function.

### 2.4. Intra-Molecular Interactions of Pdlim5 May Regulate the Accessibility of the DUF Domain

**Figure 4:**
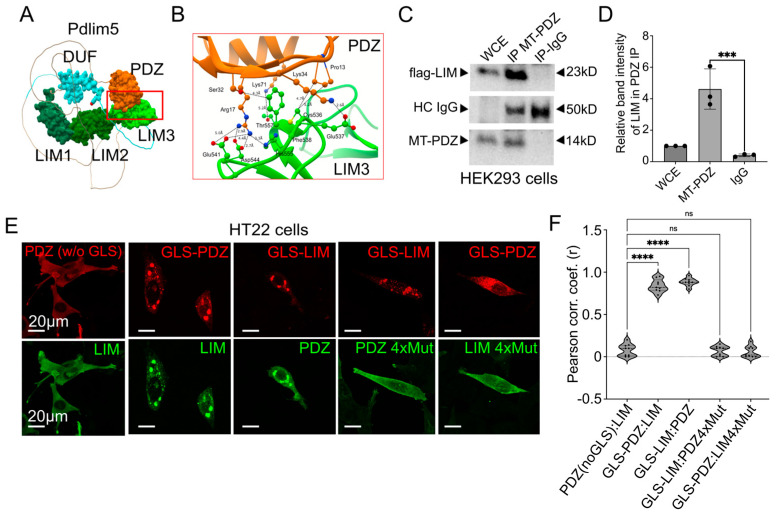
Intra-molecular interactions of Pdlim5 are suggested via IP, IF, and AlphaFold evidence. (**A**) AlphaFold predicted model of the Pdlim5 folded structure showing the PDZ (orange), DUF (cyan), and LIM (green) domains. The red box indicates a PDZ/LIM domain interface expanded in Panel (**B**). (**B**) Predicted molecular interface between the PDZ/LIM domains, identifying predicted interacting residues that form the basis of subsequent point mutations. (**C**) Following their exogenous co-expression in HEK-293 cells, flag-tagged isolated LIM domain co-precipitates with pull-downs of the isolated myc-tagged PDZ domain that are shown quantified in Panel (**D**). (**D**) Quantitation of co-immunoprecipitation band intensities from Panel (**C**), representing flag-LIM in MT-PDZ pull-down assays. Each dot represents one biological replicate. The error bars represent the SEM. The statistical significance, determined using one-way ANOVA, is indicated as *p* ≤ 0.0001 (***). (**E**) Golgi co-relocalization assays in HT-22 cells. The isolated PDZ or LIM domain of Pdlim5 when fused to a Golgi localization sequence (GLS) leads to its (red) punctate distribution at the Golgi, as expected. Experimentally, when GLS-PDZ is co-expressed with the isolated LIM domain of Pdlim5 (second image in the lower subpanel), this leads to LIM’s Golgi co-relocalization with GLS-PDZ. As predicted based on AlphaFold (Panel (**B**)), 4× point mutants of either the isolated PDZ or LIM domains fail to co-relocalize. In negative-control conditions, when PDZ lacks the GLS, it is no longer directed to the Golgi and becomes more diffuse/cytoplasmic (first image upper subpanel). In this case, the isolated LIM domain likewise no longer co-relocalizes with PDZ to the Golgi. Scale bars in the images represent 20 μm. (**F**) Quantitation of Golgi co-relocalization assays based on images as shown in Panel (**E**). Pearson’s correlation coefficient indicates co-distribution of PDZ/LIM to the Golgi under experimental conditions where either of the isolated PDZ or LIM domains is fused to a GLS. In contrast, the corresponding 4× point mutants of the isolated PDZ or LIM domains show a loss of such ectopic co-relocalization. The violin plot quartiles (dotted lines) indicate data ranges where the middle dark line represents the median. Statistical significance is determined using one-way ANOVA and indicated as *p* ≤ 0.0001 (****) or ns (not significant).

To further address Pdlim5 from a functional perspective, we investigated its intra-molecular interactions. First, we subjected Pdlim5 to the AlphaFold2 software to model potential inter-domain interactions in its predicted 3D structure (Figure 4A). Pdlim5 regions are highlighted for the PDZ (orange), DUF (cyan), and LIM (green) domains. Importantly, an interaction interface was predicted between the PDZ and LIM domains (Figure 4A), and the interfacial amino acids implicated in this PDZ/LIM association (Figure 4B) formed the basis of mutant constructs for functional tests. First, triplicate experiments of co-immunoprecipitations from cell lysates expressing myc-tagged PDZ and flag-tagged LIM domains were accomplished, and band intensities were quantified (representative blot in Figure 4C and summary data in 4D).

These results were consistent with a potential interaction between the PDZ and the third LIM domain of Pdlim5. Further support of the PDZ/LIM intra-molecular interactions came from the use of an orthogonal assay using isolated PDZ or LIM domains that are fused to a Golgi localization sequence (GLS). If two proteins associate, the partner protein will also localize to the Golgi region of transfected cells (Figure 4E). As predicted, the co-expression of the corresponding isolated putative-partner domain (not fused to GLS) demonstrated PDZ/LIM co-localization (e.g., LIM domain colocalized with GLS-PDZ). Negative controls included expressing PDZ not fused to GLS (e.g., LIM did not appear in Golgi puncta when it was co-expressed with PDZ not fused to GLS (likewise the inverse; Supplemental Figure S1). Quantitative evidence came from the application of Pearson’s correlation coefficient to assess the co-distribution of PDZ in the presence versus absence of its GLS tag (Figure 4F).

The GLS assay was then used to assess the impact of mutating the residues at the predicted contact interface between the PDZ and LIM domains identified from Alpha Fold (Figure 4B). Four contact residues were mutated from the modeled interface from each of the PDZ (PDZ4xMut; P13G, R17A, K34G, K71A) and LIM domains (LIM-4xMut; C541G, F543A, T562A, C570A). Neither the PDZ nor LIM domain mutants showed co-localization to the GLS-tagged domain of the corresponding binding partner (Figure 4E,F), suggesting that the identified residues play direct or indirect roles in forming stable contacts between the PDZ and LIM domains. Overall, our findings provide support for Pdlim5’s intra-molecular association between the PDZ and LIM domains. Thus, when we consider the dendrite branching activity promoted by Pdlim5’s central DUF domain, it is reasonable to conjecture that the PDZ/LIM interaction modulates the activity of the central DUF domain in the native full-length Pdlim5 molecule via a steric or allosteric mechanism proposed for a number of other actin-binding domains [19].

**Figure 5:**
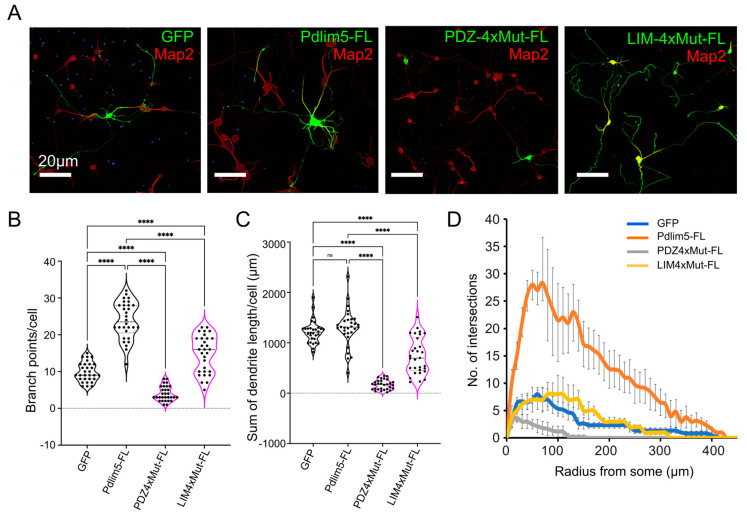
Functional effects of disrupting the PDZ/LIM intra-molecular association. (**A**) Representative images depict rat hippocampal neurons at DIV7, following transfection at DIV3 with the indicated constructs. Quantitation is shown in Panels (**B**,**C**). Neurons were visualized for exogenously expressed GFP, Pdlim5 full-length (Pdlim5-FL), Pdlim5 full-length with 4× point mutation in the PDZ domain (PDZ-4xMut-FL; P13G, R17A, K34G, K71A), or Pdlim5 full-length with 4× point mutation in the LIM domain (LIM-4xMut; C541G, F543A, T562A, C570A). Each Pdlim5-based construct was myc-tagged and was detected by immuno-labeling as described in the methodology. Immuno-labeling for Map2 is used here as a neuronal dendritic marker and is shown in red. Scale bars represent 20 mm. (**B**) Shown is the quantification of the average number of branch points per neuron following exogenous expression of GFP (negative control), Pdlim5 (positive control), or 4× point mutants within the PDZ or LIM domains of full-length Pdlim5. The choice of the 4× point-mutant residues was based on information shown in Figure 4B (AlphaFold model). Statistical significances, determined using one-way ANOVA, are indicated as *p* ≤ 0.0001 (****) and ns (not significant). (**C**) Shown is the quantification of the sum of dendrite lengths per cell, following exogenous expression of GFP (negative control), Pdlim5 (positive control), or 4× point mutants within the PDZ or LIM domains of full-length Pdlim5. Statistical significance was determined using one-way ANOVA and is indicated as *p* ≤ 0.0001 (****). (**D**) Sholl analysis of neuronal processes following the expression of GFP (negative control), Pdlim5-FL (full-length; positive-control), or the 4x point mutants within the PDZ or the LIM domain of full-length Pdlim5 (PDZ4xMut-FL or LIM4xMut-FL). Compared to GFP-expressing cells (blue line), neurons exogenously expressing Pdlim5 (orange line) significantly increase dendrite branching, and PDZ4xMut-FL (gray line) and LIM4xMut-FL (yellow line) showed a significant loss of neuronal processes (even relative to GFP). Data points are average values (*n* ≥ 15 neurons) with error bars indicating SEM. The significance was assessed using a two-way ANOVA (mixed-effect model for missing values and including repeated measures) with Bonferroni post hoc analysis that varies from *p* ≤ 0.0001 to *p* < 0.05, based on radial distance from the cell soma).

Finally, we undertook additional functional analyses using primary rat hippocampal cells to assess the impact of the mutations made at the PDZ/LIM interface on neurite growth as described in Figure 1 and Figure 2 above. In comparison to the exogenous expression of Pdlim5 full-length protein (Pdlim5-FL; positive-control), each of the two independent 4× point mutants of Pdlim5 produced significantly less branching activity (Figure 5A). This was especially the case for the 4× point mutations within the PDZ domain of Pdlim5 (PDZ4xMut-FL), where the branching activity was seen to be even less than that of the GFP negative control (Figure 5B–D). While it is important to appreciate that both such 4× point mutations may compromise protein–protein interactions occurring beyond the PDZ/LIM intra-molecular association, our results are in total consistent with the possibility that the extent of the PDZ/LIM interaction in Pdlim5 may represent a functional switch. Such a switch could conceivably also be coordinated with Pdlim5’s central DUF domain and the phospho-status of Y251 within it.

## 3. Discussion

Dendritic branching is a fundamental process in neurodevelopment, and aberrations in its execution are associated with multiple human neuronal and cognitive pathologies [21,22]. Earlier work [16] pointed to Pdlim5’s biochemical and functional association with delta-catenin, a known player in the promotion of dendritic complexity and lengthening. Mediated via the Cdk5 kinase and upstream glutamate signaling, phosphorylation of the PDZ-ligand at delta-catenin’s C-terminus enhances the association with the PDZ domain at Pdlim5’s N-terminal region and results in enhanced branching. Further, more recent findings from our group point to the C-terminal LIM domain of Pdlim5 binding to PalmD, a protein proposed to have roles in the shaping of membrane processes associated with neuronal process outgrowth [18].

In this report, we began by assessing the functional role of the three established structural regions within Pdlim5, namely the PDZ, DUF, and LIM domains, in regulating dendritic branching. Employing an in vitro culture of primary rat hippocampal neurons, this was undertaken through the exogenous expression of each of the isolated domains (e.g., isolated PDZ) or, in a complimentary fashion, through the expression of Pdlim5 constructs that lacked one of each of the three domains (e.g., PDZminus). Unexpectedly, the expression of the isolated central DUF domain of Pdlim5 exhibited potent dendrite branching activity equivalent to that of full-length Pdlim5. In contrast, the expression of the isolated PDZ domain led to more modest positive effects, and LIM to inhibitory ones. For example, LIM expression, conceivably operating in a dominant-negative capacity, reduced branching complexity to a level below that of the GFP negative control. DUF’s key role was again supported upon the expression of a full-length Pdlim5 construct lacking DUF. Compared to the deletion of either the PDZ or LIM domains, the loss of the central DUF domain displayed greater reductions in branching activity. Overall, our assays indicate that each domain is relevant to Pdlim5’s dendritic functions, as expected. Unexpectedly, however, Pdlim5’s central DUF domain appears to stand out in its relevance in regulating dendritic branching. Future mechanistic work is needed to test this assertion, for example, through the further identification or probing of protein–protein interactions that rely on each of the three domains.

In this regard, we report here the potential involvement of the phospho-residue Y251 in functions of the DUF domain. This residue is highly conserved across vertebrates, and its point mutagenesis resulted in corresponding reductions in Pdlim5’s potency in dendritic branching. Further, the Y251-peptide probing of an array of protein domains known to engage in phospho-tyrosine-dependent interactions suggested several kinases and other possible partners selective for binding the phospho-Y251 (peptide/Pdlim5) state. These phospho-specific binding proteins include Fyn, Yes, Nck2, and Abl1&2, each of which has been implicated in aspects of neuronal development. The application of AlphaFold enabled us to model phospho-Y251’s potential interaction with the middle LIM domain of Pdlim5, and it seems plausible to suggest that phosphorylation of Y251 could enhance or destabilize interactions of the DUF domain with the LIM domain. Future work will be needed to address this possibility, but conceivably, one or more of the kinases identified to bind the phospho-Y251 peptide, together with their signaling pathways, could represent an upstream switch of Y251 (Pdlim5) function or themselves instead be downstream modulators of that function.

More graphically, both AlphaFold predictions and our orthogonal biochemical and immunofluorescence cell-based tests supported the association of Pdlim5’s PDZ and LIM domains. This reflects an intra-molecular folding that brings the N- and C-terminal regions of Pdlim5 together. The implications of such folding for the functional activity of the central DUF domain are uncertain, but in the context of the full-length Pdlim5 protein, one can imagine a negative, positive, or (less likely?) neutral impact upon the capacity of DUF to promote dendritic branching. We note that PDZ-LIM domain interactions have recently also been suggested via AlphaFold2 modeling of other members of the PDLIM scaffolding family [5] and thus that this may be a general autoregulatory mechanism regulating the different members. PDLIM family proteins as noted have widespread functions including the regulation of cell differentiation, proliferation, and migration [8,23,24,25]. It is thus unsurprising that PDLIM proteins play widespread roles in organ development and tumor invasion by altering the properties of the cytoskeleton (summarized by Healy and Collins [5])

Going forward, to better understand the larger role of Pdlim5 and its partner proteins (e.g., delta-catenin, PalmD, alpha-actinin), additional structure–functional investigations will be needed into the roles played by Pdlim5’s domains. For example, this would involve identification not only of those Pdlim5 domains engaged by varied partners, but also of how those relationships might be affected by the phospho-state of Y251 in the DUF domain or the larger association state of the PDZ/LIM domains. Given that the central DUF domain’s phospho-Y251 is modeled to abut Pdlim5’s LIM domain, these two contexts may be functionally linked. For example, it is plausible that Pdlim5 possesses more than one folded state, and that even partial lessening of the PDZ/LIM association leads to positive or negative changes in the central DUF domain accessibility in promoting dendrite shaping/complexity. Finally, given that altered expression of Pdlim5, as well as delta-catenin (and PalmD), is associated with multiple neurological conditions [26,27,28,29], further study has the potential to provide an improved understanding of human pathologies.

## 4. Materials and Methods

### 4.1. Neuronal Cultures and Transfection

All vertebrate animal work was performed under the guidelines established by the UTHSC-Houston animal welfare committee under the protocol AWC-23-0082. Dissociated neuronal cultures were produced from the hippocampus and cortex of rat embryos on the 18th day of gestation (E18) as previously described [16,17]. Hippocampal neurons were plated at a density of 2 × 10^5^ cells per well into 24-well tissue-culture plates that each contained 12 mm glass coverslips that were pre-treated with 100 µg/mL poly-D-lysine (Sigma-Aldrich, St. Louis MO, USA; A-003-M). Cortical neurons were grown on 10 cm tissue-culture dishes plated at a density of 2.0 × 10^7^ cells per plate that had been pre-coated with 100 µg/mL poly-D-lysine. Neurons were plated in Opti-MEM with glucose for 1 h at 37 °C and then transferred to growth media composed of Neurobasal Medium (Life Technologies, Carlsbad, CA, USA), containing 2% B-27, GlutaMAX, and penicillin–streptomycin. On day 3 post-plating, neurons were transfected with Lipofectamine 2000 employing 1 µg plasmid and 1.5 µL of Lipofectamine in a total of 50 µL of Opti-MEM per well. At DIV7 post-plating, cells were fixed and processed for immunostaining as described below. All experiments were performed with at least three distinct biological replicates, pooling data from a minimum of 15 neurons per condition. One-way ANOVA was used to determine statistical significance.

### 4.2. HEK293 Cell Culture and Transfection

HEK293 cells from ATCC (ATCC# CRL-1573) were cultured in DMEM (Sigma-Aldrich) supplemented with 10% FBS (Sigma-Aldrich) and penicillin–streptomycin (Life Technologies). Transfection of the HEK293 cells with exogenous DNA (1 µg) was accomplished using Lipofectamine 2000 as per the manufacturer’s instructions. Transfection took place when the cells reached 50–60% confluency in six-well culture plates, and cell extracts were prepared for co-IP experiments (see below) 48 h post-transfection.

### 4.3. cDNA Constructs

All cDNA constructs (PDZ, DUF, LIM, PDZminus, DUFminus, LIMminus, etc. (please see Supplementary File “table of constructs, Table S1”)) were cloned into the backbone of the pCS2 mammalian expression vector. Engineering was undertaken by Epoch Life Science and/or the McCrea laboratory. All constructs were confirmed with DNA sequencing. Full-length human Pdlim5 was engineered previously in our laboratory [16]. DNA maxi-preps were outsourced to Epoch Life Sciences (Missouri City, TX, USA).

### 4.4. Antibodies

Commercially available antibodies that target particular epitope tags were employed. Among these were rabbit or mouse anti-Myc epitope-tag antibodies (CST #2272S and CST #9B11, respectively), rabbit or mouse anti-GFP antibodies (CST #2956 and CST #4B10), and a mouse monoclonal antibody directed against the FLAG epitope tag (Sigma #F7425). Primary mouse IgG from Invitrogen, Waltham, MA, USA (#10400C) and rabbit IgG from Life Technologies (#10500C) were used for negative control immunoprecipitations (IPs). Goat polyclonal secondary antibodies conjugated with horseradish peroxidase (HRP) were procured from Thermo Fisher Scientific, Waltham MA, USA (#31430 for anti-mouse and #31460 for anti-rabbit). Invitrogen supplied Alexa Fluor immunofluorescent secondary antibodies (A32723 and A21422 for anti-mouse, A22008 or A32732 for anti-rabbit). We used 1:1000 dilution for primary antibodies and 1:3000 dilution for secondary antibodies in co-IP protocols, while for immunohistochemistry, we used 1:1000 for primary antibodies and 1:2000 dilution for secondary antibodies.

### 4.5. Golgi Localization Assay (GLS)

Immortalized mouse hippocampal neuronal cells (HT-22) were cultured in DMEM (Sigma-Aldrich) supplemented with 10% FBS (Sigma-Aldrich) and penicillin–streptomycin (Life Technologies). Transfections of cells were performed at 50% to 60% confluency using Lipofectamine 2000 as described above for HEK-293 cells. Golgi co-relocalization was examined by fixation with 4% PFA 24 h after transfection, followed by immunostaining and subsequent image collection using confocal microscopy. To relocalize Pdlim5’s PDZ domain to the Golgi, the PDZ domain was tagged at its N-terminus with the Golgi localization sequence (GLS) of mammalian target of rapamycin (see full sequence below) [16]. Following co-transfection with Pdlim5’s C-terminal LIM domain, the cells were subjected to immunofluorescence staining as previously described [16,17]. Qualitative analysis involved visual examination of co-relocalized partner proteins to the Golgi, occurring only when GLS-PDZ was present. Using ImageJ 1.53q software (https://imagej.net/ij/), confocal images were quantitatively analyzed, and the intensity overlap between the red and green channels at the Golgi was determined. The correlation of fluorescence intensities across various optical channels was measured using Pearson’s correlation, which gives a statistical indication of how closely the intensities overlap in each condition.

GLS nucleotide sequence from mammalian target of rapamycin: 5′-ACTAGTGAAATGCTGGTCAACATGGGAAACTTGCCTCTGGATGAGTTCTACCCAGCTGTGTCCATGGTGGCCCTGATGCGGATCTTCCGAGACCAGTCACTCTCTCATCATCACACCATGGTTGTCCAGGCCATCACCTTCATCTTCAAGTCCCTGGGACTCAAATGTGTGCAGTTCCTGCCCCAGGTCATGCCCACGTTCCTTAACGTCATTCGAGTCTGTGATGGGGCCATCCGGGAATTTTTGTTCCAGCAGCTGGGAATGTTGGTGTCCTTTGTGAAGAGCCACATCAGACCTTATATGGATGAAATAGTCACCCTCATGAGA-3′.

GLS amino acid sequence from mammalian target of rapamycin: TDENAWVNMEKLPLEYSPAVSMMGPAMADIPEPSHLSSHHTMVFQAHHLHIQSPGDSNVVQFPPQVMHHRSLLTVIECVDGAIRGIFVQQAGMVVVPLVRATHQTYYDEIVTPHE.

### 4.6. Co-Immunoprecipitations and Immunoblots

For the immunoblotting assay of exogenous co-immunoprecipitated proteins, HEK293 cells were lysed 24 to 48 h following co-transfection, using established protocols [16]. In brief, following lysis (1.25 mL of 1M HEPES stock; 3.75 mL of 2M KCl stock; 37.5 μL of 2M MgCl_2_ stock; 0.25 mL of NP40 stock; 5 mL of glycerol; volume made up to 50 mL with water) and pelleting of debris, protein concentrations of lysates were equalized (500–1000 µg per IP) and incubated with primary antibodies for 2 to 12 h at 4 °C with gentle rotation. Protein-A and Protein-G Dynabeads (Invitrogen, #10002D and #10004D) were added in an equal ratio to each tube and incubated for an additional 20–30 min with gentle rotation at 4 °C. After washing, the associated proteins were eluted from the beads upon the addition of 2× sample buffer with βME and heated at 95 °C for 5 min. Co-IP samples, or in the case of whole-cell extracts, samples equalized for protein, were then loaded onto 10% polyacrylamide gels and electrophoresed on BioRad (Hercules, CA, USA) mini-PROTEAN Tetra Cells (#1658005) and run for 120 min. Proteins were then transferred to nitrocellulose membranes (GE Whatman from Thermo Fisher Scientific) using a Pierce Power Blotter. Membranes were blocked in TBS supplemented with 0.1% Tween-20 plus 1% dry milk, with incubation for 4 to 12 h at 4 °C. Blocked membranes were incubated in primary antibodies overnight at 4 °C before being incubated with HRP-conjugated secondary antibodies (Invitrogen) for 1 to 2 h at room temperature. Lastly, membranes were incubated with Pierce ECL immunoblotting substrate (Thermo Fisher Scientific) and imaged using a ChemiDoc MP imaging system (Bio-Rad). To quantitate band densities, the gel or blot was first photographed and uploaded to ImageJ, and each band to be analyzed was outlined using the selection tool. ImageJ creates intensity values for the outlined regions of interest where the area under each band’s peak reflects its intensity.

### 4.7. Immunofluorescence Staining and Confocal Microscopy

Immunostaining of rat primary hippocampal neurons or HT-22 cells employed established protocols [16,18]. Briefly, cells cultured on poly-D-lysine-coated coverslips were fixed with 4% paraformaldehyde for 10 min at room temperature and then permeabilized with 0.5% Triton X-100 solution for 15 min. Following fixation and permeabilization, cells were blocked with PBS containing 1% bovine serum albumin overnight at 4 °C. Cells were then incubated with the indicated primary antibodies overnight at 4 °C and subsequently rinsed three times with fresh PBS for 10 min each, followed by an overnight rinse with PBS at 4 °C. Cells were then incubated with Alexa Fluor fluorescent dye-conjugated secondary antibodies (Invitrogen) for 1 h at room temperature before the coverslips were washed and finally mounted onto glass slides with Vectashield (Vector Laboratories, Newark, CA, USA) mounting solution. Stained cells were visualized using a Nikon T2i Inverted Confocal Microscope with an Apo-Plan 60× 1.4 NA oil immersion objective. Images were captured with a Nikon A1-DUG GaAsP hybrid four-channel multi-detector and Nikon NIS-Elements AR 5.42.03 software (Nikon, Melville, NY, USA). All z-series images were acquired at a pixel size of 100 nm and a step size of 0.2 µm.

### 4.8. Neuronal Morphological Analysis by IMARIS9.9

IMARIS 9.9 (Oxford Instruments, Abingdon, UK) was utilized to generate a 2-dimensional maximum-projection image with background subtraction from the confocal z-series of each neuron. In cases where dendrite number was evaluated, counting focused upon the number of dendrite tips per neuron, excluding any protrusions shorter than 5 µm. Dendritic length was measured by tracing from the cell body to the tip of the dendrite using IMARIS 9.9 filament tracer. Thresholds and settings for IMARIS 9.9 filament tracer were kept the same for all samples. For Sholl analysis, the maximum-projection images were converted to binary and analyzed in IMARIS 9.9 filament tracer using concentric rings with a 5 µm step size to evaluate the number of dendrite crossings at each concentric ring in relation to the distance from the cell body [17]. We selected a 5 µm step size for sampling to provide a comprehensive assessment of dendritic branching, particularly near the cell soma. The significance of our Sholl analysis was assessed using a two-way ANOVA, specifically, a mixed-effect model that can account for repeated measures and handle missing results. The repeated nature of our measurements and any within-sample variability are adequately explained by this hypothesis. We used a Bonferroni post hoc analysis, which modifies the significance levels to account for multiple comparisons and to pinpoint certain group differences. *p*-values from *p* ≤ 0.0001 to *p* < 0.05 were found in this analysis, depending on the radial distance from the cell soma.

### 4.9. Protein Domain Array

The protein domain microarray analysis was carried out at the MD Anderson Protein Array and Analysis Core. Detailed procedures are published elsewhere [30,31]. In brief, for bacterial expression, a comprehensive library of 122 SH2 domains as well as an additional 70 PTB, PH, HYB, and C2 domains (that have the potential to bind phosphor-Y marks) was generated, and it was then cloned into the pGEX vector. After being expressed as GST fusions in Escherichia coli, these domains were purified using glutathione-Sepharose beads. Using an Aushon 2470 pin microarrayer (Aushon Biosystems, Billerica, MA, USA), the purified recombinant domains were subsequently arrayed onto glass slides coated with nitrocellulose. A GenePix 4200A Microarray Scanner (Molecular Devices Inc., San Jose, CA, USA) was used to identify the fluorescence signal after the slides were probed with biotinylated peptides that bore Y251 phosphorylated versus not phosphorylated (IVERYTEF*pY*HVPTHSDASK-biotin versus IVERYTEFYHVPTHSDASK-biotin) followed with Cy3-labeled streptavidin.

### 4.10. AlphaFold Modeling

To predict the structures of Pdlim5 protein and PDZ/LIM protein complexes, we used the AlphaFold Multimer Colab Server (https://colab.research.google.com/github/deepmind/alphafold/blob/main/notebooks/AlphaFold.ipynb#scrollTo=XUo6foMQxwS2, accessed on 31 August 2023). We started the AlphaFold prediction process after setting up the Colab environment and preparing the input data, which were in the form of amino acid sequences or pre-existing structure files in PDB format with default settings. The resulting computed protein structures as multimeric complexes were analyzed and visualized using UCSF Chimera X (https://www.cgl.ucsf.edu/chimerax/).

## Data Availability

The raw data supporting the conclusions of this article will be made available by the authors, without undue reservation. DNA constructs will also be made available upon request.

## Supplementary Materials

The following supporting information can be downloaded at: https://www.mdpi.com/article/10.3390/ijms25158326/s1.
