# Supplementary material for: Exploring the PDZ, DUF, and LIM Domains of Pdlim5 in Dendrite Branching
Source: Int J Mol Sci. 2024 Jul 30;25(15):8326. doi: 10.3390/ijms25158326 (PMC11312917; doi:10.3390/ijms25158326)
Supplement: Supplementary file 1 [file ijms-25-08326-s001.zip › ijms-3040419-supplementary.pdf]

Supplementary Figures for:

Exploring the PDZ, DUF and LIM Domains of Pdlim5 in Dendrite Branching

Yogesh Srivastava, Maxsam Donta, Lydia L. Mireles, Adriana Paulucci-Holthauzen, Leilei Shi and Mark T. Bedford, M. Neal Waxham and Pierre D. McCrea

Figure S1 – Localization of non-GLS Tagged PDZ and LIM domains.

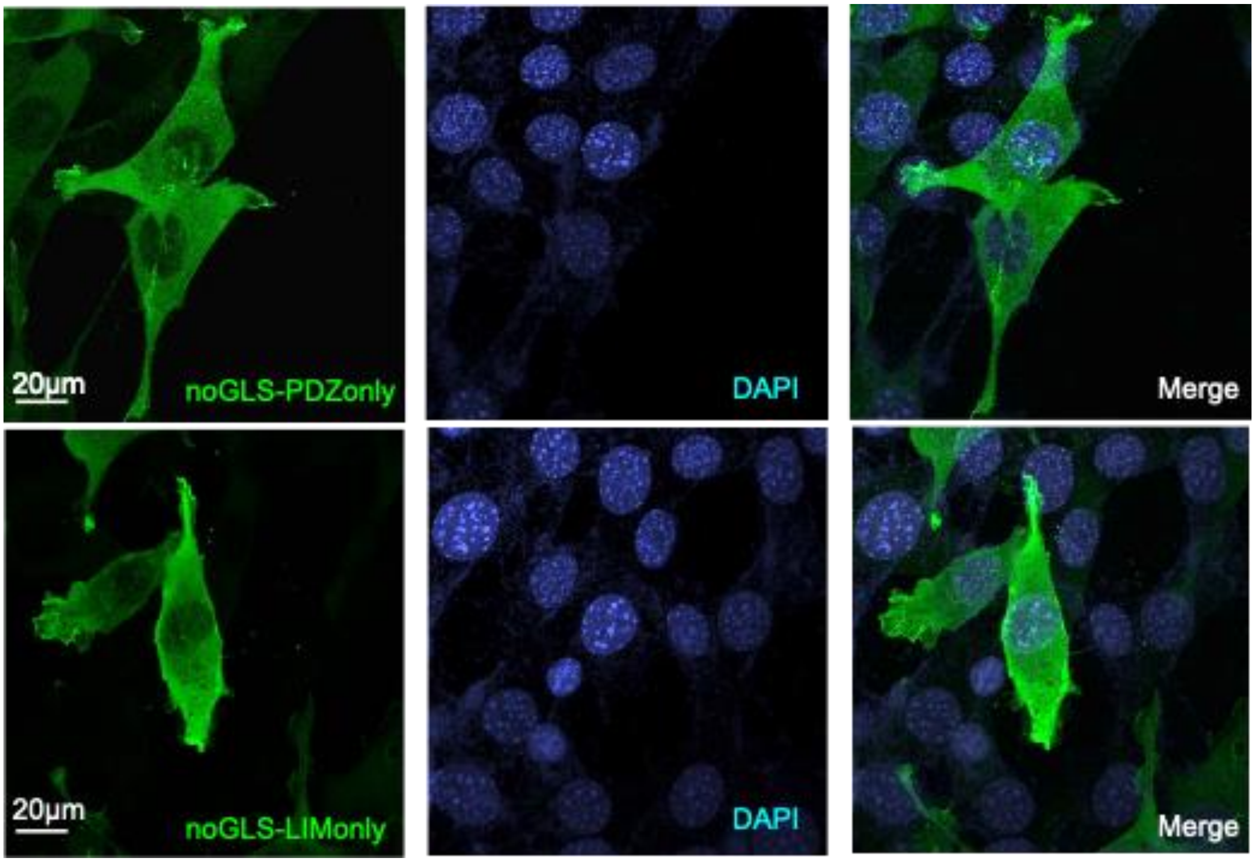

Supplementary Figure S1 – PDZ or LIM domains lacking the GLS tagging sequence were transfected into HT-22 cells. 48 h post-transfection, the cells were fixed with 4% PFA and processed for immunocytochemistry with an antibody to the Myc tag (1:1000 dilution), followed by Alexa488-labeled secondary antibody (1:2000 dilution). Immunolabeled coverslips were mounted in DAPI containing media and fluorescent images acquired on a Nikon A1 confocal microscope. Note that no Golgi localization or aggregation are evident in the transfected cells.

Figure S2 – Identification of Phosphorylated Pdlim5 when Expressed with Abl1.

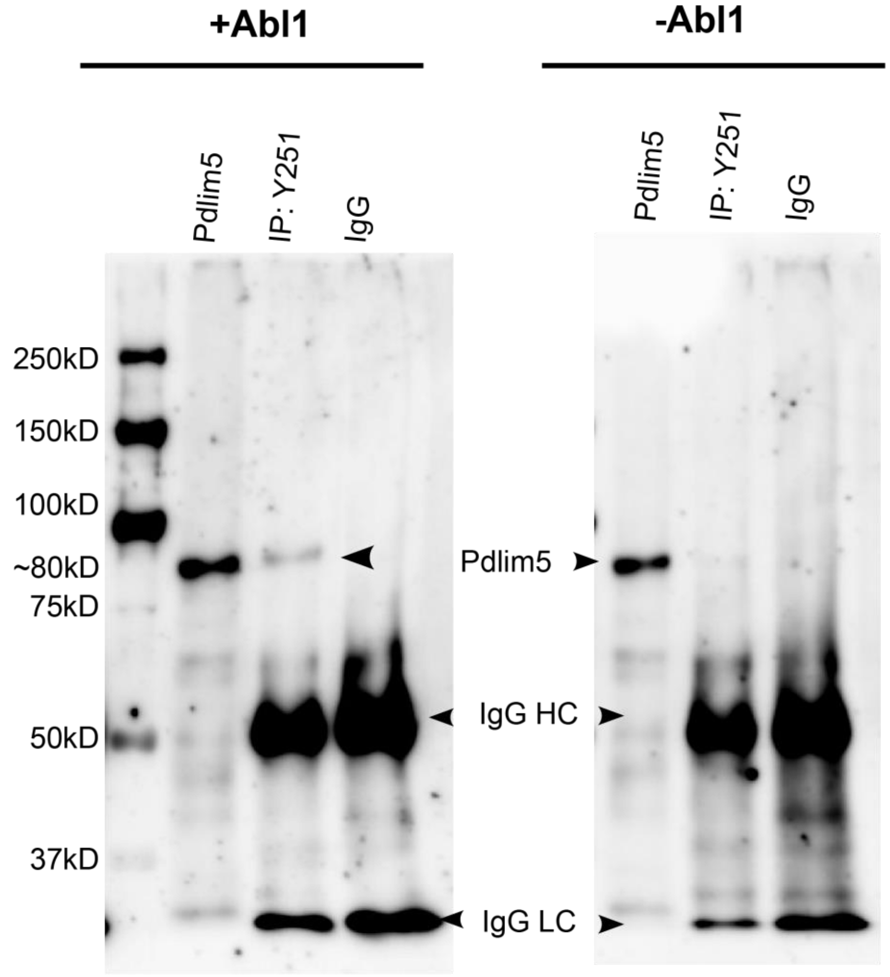

Figure S2 – Western blot of HEK293 cells transfected with MT(myc-tagged)-Pdlim5 with (+Abl1) or without (-Abl1) Abl1. Lanes labeled Pldim5 are aliquots of each extract as the loading controls for both transfections. Lanes labeled IP:Y251 are the extracts immunoprecipitated with the Phospho(Y251)-Pdlim5 antibody (PA5-40209, Invitrogen). Lanes labeled IgG represent immunoprecipitations without extract to identify the IgG heavy and light chains. The membrane was stained with an antibody to the Myc-Tag that stains Pdlim5 at ~80 kDa. The far-left lane identifies molecular weight standards ranging from 250-37 kDa. Y251-phosphorylated Pdlim5 is identified only in the blot on the left where Abl was co-transfected in HEK293 cells.

Table S1 - Table of constructs used in the manuscript

	Name of Plasmid	Construct Details	Tag
1	GFP-Pdlim5	GFP (C terminus) pCS2 vector	GFP
2	6xMT Pdlim5	6xMT (N terminus) pCS2 vector	myc-Tag
3	Pdlim5-Y251E	6xMT (N terminus) pCS2 vector	myc-Tag
4	Pdlim5-Y251I	6xMT (N terminus) pCS2 vector	myc-Tag
5	Pdlim5-PDZ-4xMut	6xMT (N terminus) pCS2 vector	myc-Tag
6	Pdlim5-LIM-4xMut	3xFlag (N terminus) pCS2 vector	flag-Tag
7	Pdlim5-LIMminus	6xMT (N terminus) pCS2 vector	myc-Tag
8	Pdlim5-LIMonly	3xFlag (N terminus) pCS2 vector	flag-Tag
9	Pdlim5-DUFonly	6xMT (N terminus) pCS2 vector	myc-Tag
10	Pdlim5-LIMonly	6xMT (N terminus) pCS2 vector	myc-Tag
11	Pdlim5-PDZonly	3xFlag (N terminus) pCS2 vector	flag-Tag
12	Pdlim5-DUFonly	3xFlag (N terminus) pCS2 vector	flag-Tag
13	Pdlim5-PDZminus	6xMT (N terminus) pCS2 vector	myc-Tag
14	Pdlim5-LIMminus	6xMT (N terminus) pCS2 vector	myc-Tag
15	Pdlim5-PDZonly	6xMT (N terminus) pCS2 vector	myc-Tag
16	Pdlim5-DUFminus	6xMT (N terminus) pCS2 vector	myc-Tag
17	Pdlim5-PDZ-4xMut-FL	6xMT (N terminus) pCS2 vector	myc-Tag
18	Pdlim5-LIM-4xMut-FL	3xFlag (N terminus) pCS2 vector	flag-Tag
19	GLS-Pdlim5-FL	6xMT (N terminus) pCS2 vector	myc-Tag
20	GLS-PDZminus	6xMT (N terminus) pCS2 vector	myc-Tag
21	GLS-PDZonly	6xMT (N terminus) pCS2 vector	myc-Tag
22	GLS-DUFonly	6xMT (N terminus) pCS2 vector	myc-Tag
23	GLS-LIMonly	6xMT (N terminus) pCS2 vector	myc-Tag
24	GLS-Pdlim5-PDZ-4xMut	6xMT (N terminus) pCS2 vector	myc-Tag
25	GLS-LIMminus	6xMT (N terminus) pCS2 vector	myc-Tag
26	GLS-Pdlim5-Y251E	6xMT (N terminus) pCS2 vector	myc-Tag
27	GLS-Pdlim5-Y2511I	6xMT (N terminus) pCS2 vector	myc-Tag

GLS – Golgi Localization Sequence; MT – Myc tag; Flag – Flag tag
All constructs were constructed in the pCS2 vector that drives expression from the CMV IE94 promoter.
